# Refractory seizures as a manifestation of anti-epileptic drug induced hypocalcemia and vitamin D deficiency

**DOI:** 10.1210/jcemcr/luag126

**Published:** 2026-05-14

**Authors:** Angela Gallucci, Jennifer Bolick, Franklin Thelmo

**Affiliations:** Christiana Care Hospital, Department of Internal Medicine, Newark, DE 19718, USA; Christiana Care Hospital, Department of Internal Medicine, Newark, DE 19718, USA; Christiana Care Hospital, Department of Endocrinology, Newark, DE 19718, USA

**Keywords:** hypocalcemia, seizure, vitamin d deficiency, antiepileptic medication, status epilepticus

## Abstract

Enzyme-inducing antiepileptic drugs (EIAEDs) such as carbamazepine, phenytoin, phenobarbital, and primidone are used in the management of epilepsy and can alter hepatic enzymes of the cytochrome P450 (CYP450) system. This can have a significant impact on vitamin D metabolism. We describe the case of a 34-year-old female diagnosed with focal epilepsy who presented to the hospital at 25-weeks gestation with recurrent seizures. On admission, she had multiple generalized tonic-clonic seizures with nystagmus requiring repeated rounds of intravenous (IV) medications. The patient was intubated and sedated and brought for emergency caesarean section due to persistent fetal decelerations in the setting of refractory status epilepticus. Laboratory investigations revealed a serum calcium level of 5.30 mg/dL (SI: 1.32 mmol/L) (reference range, 8.50-10.50 mg/dL [SI: 2.12-2.62 mmol/L]). The patient required numerous antiepileptic infusions for seizure suppression. Further evaluation revealed low 25-hydroxy vitamin D, suggesting significant hypocalcemia and vitamin D deficiency culminating in super-refractory focal status epilepticus due to chronic EIAED use. This case underscores the need to maintain a high index of suspicion for metabolic disturbances in patients on long-term EIAED therapy with regular monitoring of vitamin D and calcium levels to prevent complications.

## Introduction

Enzyme-inducing antiepileptic drugs (EIAEDs) such as carbamazepine, phenytoin, phenobarbital, and primidone are commonly used in the management of epilepsy. These medications exert their therapeutic effects in part by enhancing the activity of various hepatic enzymes, most notably those of the cytochrome P450 (CYP450) system [1]. While this enzymatic induction is beneficial for seizure control, it also has significant implications for the metabolism of other compounds, including vitamin D [1-3].

Vitamin D plays a critical role in calcium homeostasis primarily by enhancing calcium absorption in the intestines through increased expression of calcium-binding proteins and calcium transport channels in the intestinal epithelium [2]. Without sufficient active vitamin D (1,25-dihydroxyvitamin D), these transport mechanisms are downregulated, impairing the efficiency of calcium uptake from the gastrointestinal tract. Without adequate vitamin D, absorption of dietary calcium is markedly reduced [1, 2]. In a vitamin D sufficient state, calcium absorption can be augmented by 30-40% [2].

Hypocalcemia can result in a variety of clinical symptoms. These may include neuromuscular dysfunction (such as muscle cramps, tetany, or spasms), numbness or tingling in the fingers and around the mouth, fatigue, irritability, and in more severe cases, seizures or cardiac arrhythmias [4]. These symptoms reflect the critical role calcium plays in nerve conduction, muscle contraction, and cardiac function [4]. The enzyme-inducing properties of certain antiepileptic drugs can significantly disrupt vitamin D metabolism and calcium balance, posing a risk not only to bone health but also to neuromuscular and cardiovascular stability.

## Case presentation

A 34-year-old female with a history of focal epilepsy diagnosed during childhood, complicated by status epilepticus and chronic hyponatremia, presented to the hospital at 25-weeks gestation with complaints of recurrent seizures over the last several weeks. The patient reported a total of 5 seizures on the day of presentation to the emergency department. She initially denied contractions, loss of fluid, vaginal bleeding, urinary symptoms, headache, and visual changes. She reported normal fetal movement prior to arrival. Upon admission to the hospital, the patient had a witnessed generalized tonic-clonic seizure while still in the emergency room. She proceeded to have multiple witnessed generalized tonic-clonic seizures with nystagmus. She returned to baseline after each seizure and was able to communicate and follow commands between episodes. Each seizure lasted approximately 90 seconds, with some reaching 2 minutes. A rapid response was called by the admitting physician to expedite care in the setting of recurrent seizures. The patient was initially able to provide some collateral information; however, her mental status worsened as her seizures persisted.

## Diagnostic assessment

The patient further decompensated during the course of the rapid response. On physical exam, she no longer opened her eyes to verbal stimuli and was unable to follow commands. She exhibited a dysconjugate gaze, had no corneal reflex, and no cough reflex. Strength was documented as 0/5 in all 4 extremities. Her blood pressure was 112/61 mmHg, heart rate 123 beats per minute, and temperature 37.2 degrees Celsius.

Pertinent laboratory data are shown in Table 1. Notable labs include serum sodium of 154 mEq/L (SI: 154 mmol/L) (reference range, 136-145 mEq/L [SI: 136-145 mmol/L]) and calcium of 5.30 mg/dL (SI: 1.32 mmol/L) (reference range, 8.50-10.50 mg/dL [SI: 2.12-2.62 mmol/L]) with normal renal function, glucose, and no hypomagnesemia.

**Table 1:** Laboratory values the first 24 hours of admission

	Measured value	Reference range
Metabolic panel		
Sodium	154 mEq/L (154 mmol/L)	136-145 mEq/L (136-145 mmol/L)
Potassium	4.20 mEq/L (4.20 mmol/L)	3.50-5.00 mEq/L (3.50-5.00 mmol/L)
Chloride	126 mEq/L (126 mmol/L)	98-107 mEq/L (98-106 mmol/L)
Bicarbonate	19 mEq/L (19 mmol/L)	22-29 mEq/L (22-29 mmol/L)
Glucose	149 mg/dL (8.27 mmol/L)	74-99 mg/dL (4.12-5.49 mmol/L)
Blood urea nitrogen	3 mg/dL (1.07 mmol/L)	8-21 mg/dL (2.86-7.50 mmol/L)
Creatinine	0.67 mg/dL (59.20 µmol/L)	0.50-1.20 mg/dL (44.20-106.08 µmol/L)
Albumin	2.60 g/dL (26 g/L)	3.80-5.10 g/dL (38-51 g/L)
Phosphorus	1.50 mg/dL (0.48 mmol/L)	2.50-4.50 mg/dL (0.81-1.45 mmol/L)
Magnesium	3.90 mg/dL (1.60 mmol/L)	1.70-2.40 mg/dL (0.70-0.99 mmol/L)
Calcium	5.30 mg/dL (1.32 mmol/L)	8.50-10.50 mg/dL (2.12-2.62 mmol/L)
Ionized calcium	3.84 mg/dL (0.96 mmol/L)	4.48-5.28 mg/dL (1.12-1.32 mmol/L)
Aspartate aminotransferase	49 U/L (0.82 µkat/L)	11-39 U/L (0.18-0.65 µkat/L)
Alanine aminotransferase	27 U/L (0.45 µkat/L)	7-52 U/L (0.12-0.87 µkat/L)
Alkaline phosphatase	53 U/L (0.88 µkat/L)	50-135 U/L (0.83-2.25 µkat/L)
Total bilirubin	0.20 mg/dL (3.42 μmol/L)	0.20-1.00 mg/dL (3.42-17.10 μmol/L)
Complete blood count		
White blood cells	14 500 ×10^3^/µL (4.50 ×10^9^/L)	3500-11,000 ×10^3^/µL (3.50-11.00 ×10^9^/L)
Hemoglobin	8.10 g/dL (81 g/L)	11.70-15.70 g/dL (117-157 g/L)
Hematocrit	23.90% (0.24 L/L)	35-47% (0.35-0.47 L/L)
Mean corpuscular volume	89.50 fL	80-100 fL
Platelets	271 000 ×10^3^/µL (271 ×10^9^/L)	150,000-400,000 ×10^3^/µL (150-400 ×10^9^/L)
Endocrinology		
25-Hydroxy vitamin D	18 ng/mL (45 nmol/L)	>27 ng/mL (>67.5 nmol/L)
1,25-Hydroxy vitamin D	14 pg/mL (33.60 pmol/L)	18-72 pg/mL (43.20-172.80 pmol/L)
Parathyroid hormone (1 week after hospitalization)	57 pg/mL (6.04 pmol/L)	15-65 pg/mL (1.59-6.89 pmol/L)
Thyroid-stimulating hormone	0.21 mIU/L	0.27-4.20 mIU/L
Free thyroxine	1.20 ng/dL (15.48 pmol/L)	0.90-1.70 ng/dL (11.61-21.93 pmol/L)

The patient was initially administered 4 g of intravenous (IV) magnesium, 6 mg of IV lorazepam, and 4 g of IV levetiracetam. She also received several doses of 3000 mg of IV calcium gluconate supplementation. Despite this, seizure activity continued, necessitating additional doses of lorazepam (2 mg IV). The patient subsequently underwent endotracheal intubation and was sedated with a propofol infusion for the management of refractory seizures. Fetal monitoring revealed persistent decelerations. The obstetrics team was promptly called to the bedside, and the patient was taken for emergency caesarean section due to non-reassuring fetal heart tracings.

The patient was admitted to the neurological intensive care unit (ICU) postoperatively and placed on a continuous electroencephalogram (cEEG). An evaluation for secondary causes of seizures was conducted. Laboratory results remained notable for persistent, severe hypocalcemia of unknown origin. Preeclampsia labs were within normal parameters and consulting specialists considered eclampsia to be an unlikely diagnosis.

The patient presented with hypernatremia at the time of admission, which normalized within 48 hours. She continued to experience seizures with normal sodium levels. There was no hypoglycemia, renal dysfunction, uremia, substance withdrawal, or head trauma that could account for seizure activity.

A non-contrast computerized tomography (CT) scan of the head demonstrated no acute intracranial pathology. Magnetic resonance imaging (MRI) of the brain with contrast similarly showed no acute intracranial findings but did reveal a new punctate enhancement in the left medial orbital gyrus of indeterminate significance and inflammatory changes within the paranasal sinuses. Lumbar puncture was negative for infection. Further infectious evaluation revealed negative urinalysis and blood cultures. The sputum culture was positive for moderate *Haemophilus influenzae*. Initial empiric therapy with piperacillin/tazobactam 4.50 g IV every 6 hours was transitioned to ceftriaxone 2 g IV daily for pneumonia. Following intubation, endotracheal cultures identified *Acinetobacter baumannii* and *Klebsiella pneumoniae*, prompting a switch to cefepime 2 g IV every 12 hours. The patient subsequently tested positive for *Clostridium difficile* (*C. difficile*) toxin B protein gene by polymerase chain reaction (PCR) and by enzyme immunoassay. She was transitioned to vancomycin 125 mg 4 times daily for *C. difficile* infection and cefepime was discontinued. Persistent watery diarrhea likely contributed to ongoing electrolyte disturbances and hypocalcemia.

## Treatment

Her treatment regimen at this time included a propofol drip, levetiracetam 1000 mg IV twice daily, and oxcarbazepine 1350 mg IV twice daily. Hospital course was complicated by persistent status epilepticus despite multiple antiepileptic medications and sedation. She continued to have electrographic seizures and was reloaded with levetiracetam 2 g IV with an increase of maintenance dose to 1500 mg IV twice daily. The patient was also loaded with 20 mg/kg of fosphenytoin and 18 mg/kg of phenobarbital and started on maintenance doses of fosphenytoin 100 mg IV 3 times daily and phenobarbital 64.80 mg IV 3 times daily. Phenytoin and phenobarbital serum levels remained in an appropriate range at 19.60 mg/L (SI: 77.69 μmol/L) (reference range, 10-20 mg/L [SI: 39.64-79.28 μmol/L]) and 20.10 mg/L (SI: 86.55 μmol/L) (reference range, 15-40 mg/L [SI: 64.59-172.24 μmol/L]), respectively. Ketamine and midazolam drips were initiated and titrated for seizure suppression as cEEG displayed persistent status epilepticus. The patient required close monitoring of electrolytes with repletion when indicated. She received calcium gluconate IV at doses of 1000 and 2000 mg as needed on numerous occasions. Vitamin D3 supplementation of 7000 units daily via nasogastric tube was initiated.

**Figure 1:**
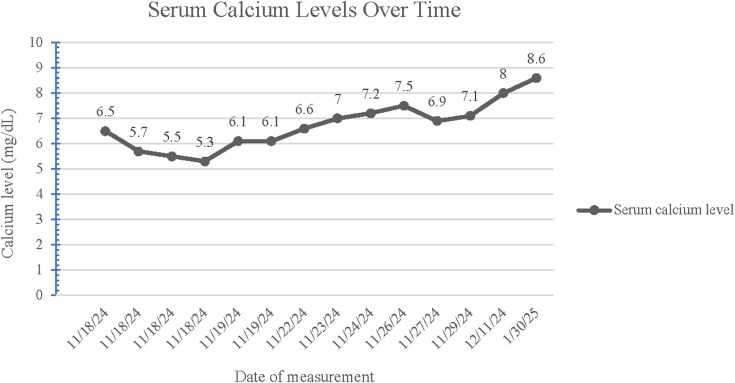
Trend of serum calcium levels as measured via metabolic panel during and after hospitalization.

Persistent seizures prompted further revision of her regimen. Oxcarbazepine was discontinued and she was subsequently loaded with lacosamide 200 mg IV and perampanel 16 mg IV. In addition, levetiracetam was switched to brivaracetam, and topiramate was added to optimize seizure control. With stabilization of calcium levels (demonstrated in Fig. 1), the patient was transitioned to calcium carbonate 500 mg 3 times daily via nasogastric tube and then orally upon extubation. After several days, the patient ultimately stabilized and was extubated. She remained seizure free with stabilization of calcium levels. Following an extensive diagnostic workup, persistent hypocalcemia was identified as the underlying cause of the patient's recurrent seizures.

## Outcome and follow-up

**Figure 2:**
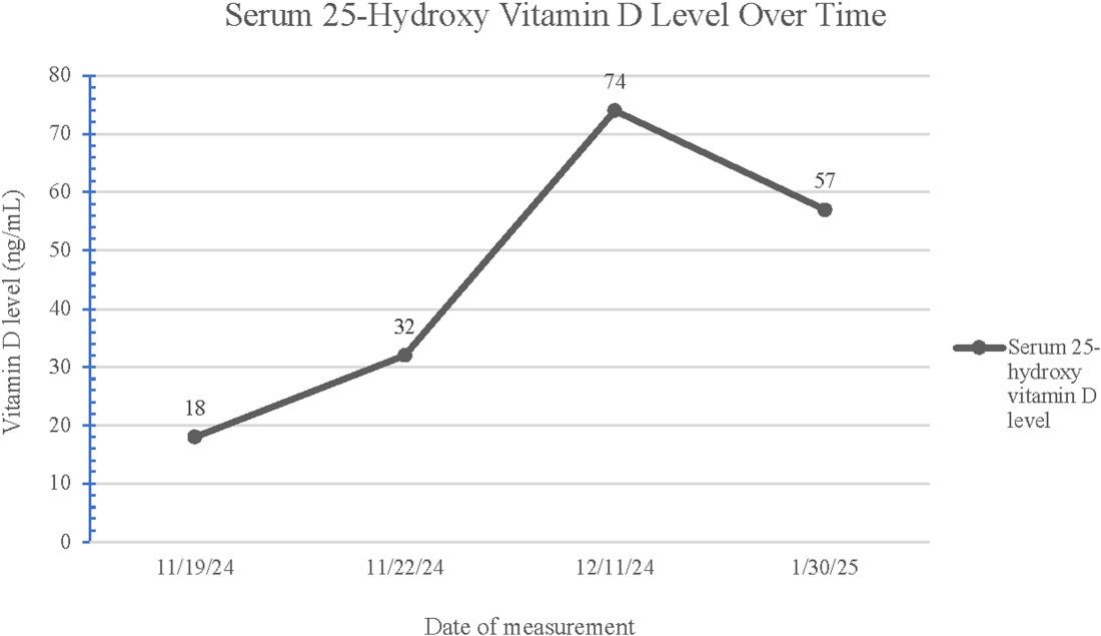
Serum 25-hydroxy vitamin D trended during and after hospital admission.

At the patient's most recent follow-up visit, her serum calcium level was 8.90 mg/dL (SI: 2.22 mmol/L) with an ionized calcium of 5.00 mg/dL (SI: 1.25 mmol/L) (reference range, 4.48-5.28 mg/dL [SI: 1.12-1.32 mmol/L]). A 24-hour urine calcium test was within normal limits. Figure 2 demonstrates monitoring of vitamin D levels over time. To help prevent further episodes of hypocalcemia, she was started on calcitriol 0.50 mcg daily. She completed a genetic evaluation that identified a variant associated with aggressive seizures that are challenging to control.

## Discussion

This case highlights a rare presentation of super-refractory focal status epilepticus, ultimately attributed to persistent hypocalcemia secondary to vitamin D deficiency from antiepileptic medications. For over 13 years, this patient was treated with various combinations of high dose antiepileptics including carbamazepine, oxcarbazepine, fosphenytoin, tiagabine, zonisamide, phenobarbital, lacosamide, perampanel, and bivaracetam. It was previously unknown why this patient required an extensive antiepileptic regimen until recent genetic testing revealed a predisposition to aggressive seizures. Lifelong antiepileptic treatment likely brought forth this severe presentation. According to multiple outpatient neurology notes, the patient's chronic hyponatremia was also attributed to her antiepileptic medications. She experienced no reported complications and was monitored with metabolic panels.

The pathophysiology of EIAED induced vitamin D deficiency and hypocalcemia is complex. EIAEDs have been associated with reduced vitamin D levels due to upregulated hepatic CYP450 enzymes that accelerate the catabolism of vitamin D [1]. This enzymatic activity increases the degradation of both 25-hydroxyvitamin D and its active form, 1,25-dihydroxyvitamin D, thereby impairing intestinal calcium absorption and contributing to hypocalcemia [2, 3]. The CYP450 enzymes are also involved in the hydroxylation steps required to form 1,25-dihydroxyvitamin D [3]. EIAEDs such as phenytoin can directly act as agonists of renal catabolic enzymes that cleave 25-hydroxyvitamin D and 1,25-dihydroxyvitamin D into their inactive metabolites [1, 3].

Vitamin D plays an important role in calcium homeostasis by promoting the expression of calcium-binding proteins and transporters in the intestinal epithelium, thereby enhancing calcium uptake from the gastrointestinal tract [2, 4, 5]. Upon synthesis in the skin or ingestion through the diet (as D2 or D3), vitamin D is transported to the liver, where it is hydroxylated at the 25-position to form 25-hydroxyvitamin D [2]. This intermediate is then converted in the kidneys to 1,25-dihydroxyvitamin D, the hormonally active form that exerts its effects by binding to vitamin D receptors in target tissues [2].

In a vitamin D deficient state, intestinal calcium absorption may drop significantly, often resulting in hypocalcemia [5, 6]. This imbalance can increase neuronal excitability and lower the seizure threshold [6]. In this case, persistent hypocalcemia from EIAED induced vitamin D deficiency culminated in super-refractory focal status epilepticus despite appropriate antiepileptic dosing. Unfortunately, we do not have results of antiepileptic blood levels obtained at this patient's first point of contact with the healthcare system, though we were able to locate a normal serum oxcarbazepine level of 30 μg/mL (SI: 118 μmol/L) (reference range, 10-35 μg/mL [SI: 40-140 μmol/L]) months prior to presentation.

Although hypocalcemia is a recognized contributor to seizure susceptibility, its role in prolonged or super-refractory status epilepticus is not as well documented. The failure of multiple antiepileptic medications to control this patient's seizures highlights the importance of identifying and correcting underlying metabolic abnormalities. The use of calcitriol (1,25-dihydroxyvitamin D) was particularly important in the management of this patient as it bypasses hepatic activation, thereby providing a direct means of restoring active vitamin D levels and improving calcium absorption [7].

This case underscores the need for clinicians to maintain a high index of suspicion for metabolic disturbances in patients with refractory seizures, particularly those on long-term EIAED therapy. Routine monitoring of vitamin D and calcium levels should be considered in this high-risk population.

## Learning points

- Know the enzyme-inducing antiepileptic drugs (carbamazepine, phenytoin, phenobarbital, and primidone).
- EIAEDs can enhance activity of cytochrome P450 (CYP450) system, which affects metabolism of vitamin D.
- Enhanced activity of CYP450 system increases degradation of 25-hydroxyvitamin D and 1,25-dihydroxyvitamin D, which impairs intestinal calcium absorption, contributing to hypocalcemia.
- Hypocalcemia may have potentially fatal complications such as seizures, cardiac arrhythmias, and heart failure.

## Contributors

All authors made individual contributions to authorship. A.G. conceptualized the case report and was primarily responsible for interpretation and acquisition of data and manuscript preparation for all sections. F.T. was involved in the diagnosis and management of the patient. J.B. assisted with patient management, data analysis, and generation of tables and figures. All authors reviewed and approved the final draft.

## Data Availability

Data sharing is not applicable to this article as no datasets were generated or analyzed during the current study.
